# Does Cognitive Training Improve Mobility, Enhance Cognition, and Promote Neural Activation?

**DOI:** 10.3389/fnagi.2022.845825

**Published:** 2022-05-23

**Authors:** Uros Marusic, Joe Verghese, Jeannette R. Mahoney

**Affiliations:** ^1^Institute for Kinesiology Research, Science and Research Centre Koper, Koper, Slovenia; ^2^Department of Health Sciences, Alma Mater Europaea—ECM, Maribor, Slovenia; ^3^Department of Neurology, Albert Einstein College of Medicine, Bronx, NY, United States; ^4^Department of Medicine, Albert Einstein College of Medicine, Bronx, NY, United States

**Keywords:** visual evoked potentials (VEP), motor-related cortical potentials (MRCP), executive control, cognitive-motor brain networks, healthy aging, sensorimotor integration, functional mobility

## Abstract

A close inter-relationship between mobility and cognition is reported in older adults, with improvements in gait performance noticeable after cognitive remediation in frail individuals. The aim of this study was to evaluate the efficacy of computerized cognitive training (CCT) on mobility in healthy, independently living older adults, and to determine whether CCT is associated with changes in neural activation for mobility-related brain processes. Using a randomized single-blind control design, sixty-three non-demented adults age 60 y and older (mean age = 67 y; 76% female, mean Montreal Cognitive Assessment [MoCA] score = 27) were recruited from a local Senior Activity Center. Participants were randomly assigned to either a 2-month CCT program (8 weeks, 3x/week, 40 min/session) or a wait-list control group. Primary outcome was self-selected gait speed during single- and dual-task walking. Secondary outcome was executive function on Trail Making Test (TMT), Part B. Neural activity was assessed *via* electroencephalography/event-related potentials (EEG/ERPs) targeting lower-limb performance. Results from a linear mixed effect model, adjusted for baseline MoCA score, age, gender, and study completion revealed that compared to controls, CCT improved gait speed during the dual-task (*p* = 0.008) but not during the single-task walking condition (*p* = 0.057). CCT also improved executive function (*p* = 0.024). Further, shorter foot reaction time responses (*p* = 0.019) were found with enhanced neural activation over sensorimotor areas, with shorter ERP latencies during the P2 component (*p* = 0.008) and enhanced motor responses (*p* = 0.009) also evident in the CCT group after the intervention. Overall, the electrophysiological findings suggest possible neural adaptations that could explain improvements in mobility and executive functions associated with CCT in healthy older adults.

## Introduction

Walking difficulties are widespread among older adults, and are associated with restricted activities of daily living, mobility disability and death (Hirvensalo et al., 2000; Rosano et al., 2008). While exercise is recommended to improve mobility (Urzi et al., 2019), only 16% of U.S. seniors exercise at the recommended level and 33% remain inactive (Lee et al., 2017). Sedentary behavior or inactivity is related to increased risk of disability within all levels of physical activity (DiPietro et al., 2018).

Among non-pharmacological interventions, cognitive training has been shown to stabilize or even improve cognitive performance of healthy older adults (Tardif and Simard, 2011; Simons et al., 2016). Gait and cognitive functions are interrelated in older adults. Previous studies have revealed that the prefrontal cortex (PFC) plays a critical role in successful gait and cognition (Beauchet et al., 2016) and have linked gait to structural changes in cerebellar, precuneus, supplementary motor, insular, and PFC brain regions (Blumen et al., 2019). These findings support the notion that cognitive training could improve both cognitive and motor functions. Additionally, previous experiments have shown that cognitive training can improve response times, with improvements seen in multiple ERP components in healthy young (Olfers and Band, 2018) and old adults (Gajewski and Falkenstein, 2012, 2018; Pergher et al., 2018), promoting neural enhancements in aging (Nguyen et al., 2019).

Impairments in cognitive processes are associated with slower gait and gait instability (Montero-Odasso et al., 2012). Successful mobility performance is dependent on intact cognitive function (Montero-Odasso et al., 2012). Hence, recent research has focused on ways to improve cognitive function as a means to improve mobility. In a pilot study with sedentary seniors, Verghese et al. (2010) reported a far transfer computerized cognitive training (CCT) effect to mobility. A recent meta-analysis of 10 trials with 351 participants reported that cognitive training interventions can improve mobility-related outcomes, especially during challenging walking conditions requiring higher-order executive functions (Marusic et al., 2018a,b). More recently, computerized cognitive remediation improved walking and executive functions in older adults aged 70 years and above at high-risk for mobility disability, but not compared to an active control that included low level cognitive remediation training (Verghese et al., 2021).

Most studies examining the effect of CCT on mobility-related outcomes enrolled sedentary or frail older adults (Verghese et al., 2010; Smith-Ray et al., 2013; Ng et al., 2015; Azadian et al., 2018), and demonstrate significant CCT effects on mobility outcomes. Although the association between brain health, cognition and mobility has been demonstrated even in non-clinical populations (Cohen et al., 2016; Demnitz et al., 2017), the effect of CCT in healthy active older adults has not been established. Moreover, the underlying neural mechanisms of enhanced gait control after cognitive remediation programs have not been well investigated (Marusic et al., 2018b).

To address these knowledge gaps, we conducted a randomized single-blind control trial in 63 healthy active older adults. Our hypotheses were based on our previous work (Marusic et al., 2018a,b), which indicated that CCT would lead to improvements in executive functions and gait performance, particularly during challenging walking while talking conditions. We also hypothesized that CCT-related enhancements would be evident in pre-post assessments using electroencephalography (EEG)/event-related potentials (ERPs) over sensorimotor regions. This was guided by our recent investigation of the efficiency of recruitment of neuronal resources for the upper and lower extremity response task in young and old adults (Marusic et al., 2022), thus sparking an interest in investigating CCT related changes in neural activation.

## Methods

### Study Design

The current study employed a single-blind randomized control trial design to investigate the efficacy of computerized cognitive training (CCT) on gait in healthy older adults recruited from a local adult activity center in Koper, Slovenia (EU). The inclusion criteria were: active and healthy older adults (defined according to self-reported questionnaires), normal or corrected normal vision, and no self-reported history of cardiovascular, neurological, or psychiatric conditions. The exclusion criteria were: symptoms of cognitive decline or evidence of cognitive impairment on the Montreal cognitive assessment (MoCA; score < 25), history of falls in the past 12 months, regular heavy alcohol consumption, or presence of acute or chronic skeletal, neuromuscular, metabolic and/or cardiovascular disease conditions that may limit mobility. All procedures were carried out in accordance with the ethical standards of the 1964 Declaration of Helsinki and were approved by the Republic of Slovenia National Medical Ethics Committee (KME57/06/17). Written informed consent was obtained from all participants prior to study enrollment. The trial was registered at ClinicalTrials.gov (NCT03860441).

### Participants

**Figure 1:**
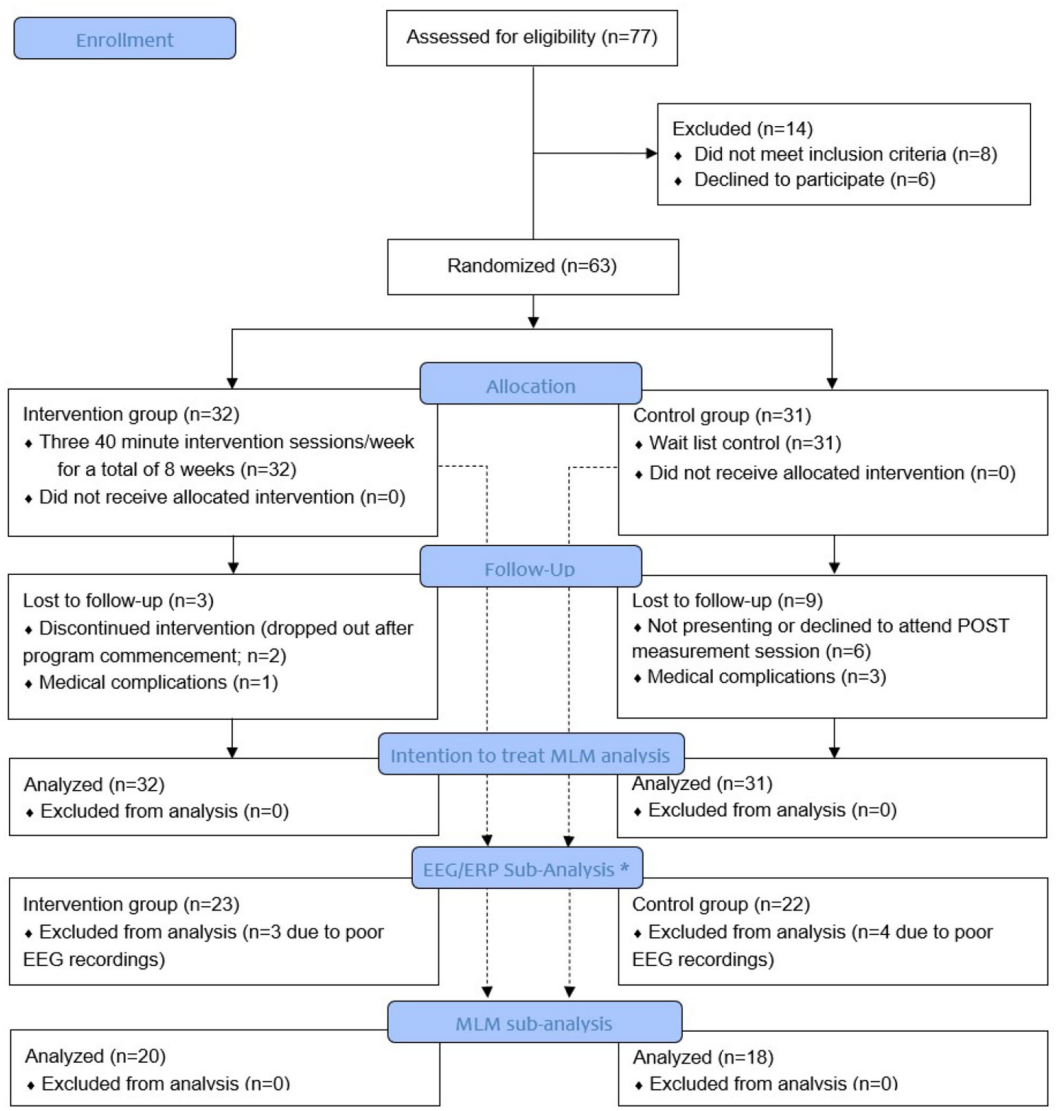
Study flow diagram. *A mixed linear model (MLM) analysis of variance was used for EEG sub-analysis when EEG data were of good quality and a merged pre-post EEG decomposition was acceptable. Poor quality of pre- or post-EEG data resulted in elimination of both datasets.

Figure 1 presents the study flow. From the initial 77 participants who were assessed for eligibility, 63 were randomized to either the intervention or the wait-list control group. Fourteen participants were excluded because they either did not meet study criteria or were unwilling to participate in the study. Sixty-three older participants (67.5 ± 5.9 years; MoCA score: 27.2 ± 1.7) were enrolled and included in the intention to treat analyses (see Figure 1). A subsample of 45 participants agreed to complete an EEG / ERPs study before and after the CCT intervention.

#### Clinical Assessments

Prior to the intervention, all participants were screened and interviewed by the research team. We asked participants to rate their general health on a 5-point Likert scale ranging from 1 (worst possible) to 5 (best). As previously mentioned, the MoCA (Nasreddine et al., 2005) was used to determine inclusion eligibility and also served as a covariate in the statistical models.

#### Computerized Cognitive Training Intervention

A commercially available internet-based cognitive training software CogniFit (CogniFit INC; San Francisco, CA, USA) was used for training. CogniFit software provides participants' with individualized training programs based on the results of their baseline cognitive performance evaluation. Difficulty of each cognitive task or game is systematically increased by the software in order to gradually increase the difficulty of each subsequent task, while ensuring that all abilities are trained. As well, this methodology is intended to provide personalized programs that strategically strengthen the individual's weakest cognitive abilities by emphasizing cognitive strengths in a manner that keeps participants within their comfort zone. The CCT training software in this study (CogniFit) is identical to that employed by Verghese et al. (2010, 2016, 2021) in their previous cognitive remediation trials.

Participants in the CCT group attended 40 mins training sessions three times weekly for a total of 8 weeks (24 sessions). The length of each daily session was set according to optimal duration determined in our systematic review (Marusic et al., 2018b). Sessions were administered at the activity center. CCT sessions included a maximum of eight participants and a minimum of two research assistants who aided with software initiation and insured participants' comprehension of each CCT task instruction set.

#### Wait-List Control

Participants randomly allocated to the control group were informed that upon conclusion of baseline measurements, their name would be added to a wait-list for future studies at our center. They received a lecture detailing the importance of maintaining a healthy lifestyle. Research assistants contacted control participants weekly *via* telephone to maintain interest and adherence in the trial as well as to match for study contacts in the intervention arm. Participants in the wait-list group were not revealed to the intervention group participants, and vice versa as members of each group were invited to the activity center on different days and/or times.

#### Outcome Measures

Our primary outcome measure was gait speed (m/s) during single- and dual-task walking conditions. Our secondary outcomes included executive function performance as assessed with the Trail making test (TMT); a simple visual reaction time task with lower limb as part of the EEG study; as well as sensory and motor ERPs. Research assistants assessed all outcome measures blinded to study group allocation at baseline and immediately following the conclusion of the 8-week study period for both the intervention and control groups.

#### Single- and Dual-Task Walking

Gait speed in single and dual task conditions was selected as our primary outcome as it is the most widely used metric to characterize mobility, and it was demonstrated to be sensitive to CCT effects (Verghese et al., 2010). Participants were instructed to “walk as if they were going to the nearest store and they were not in any hurry.” Gait speed was acquired with the 2D OptoGait system (Microgate, Bolzano, Italy); a valid and reliable measure of gait (Lienhard et al., 2013). To measure steady-state gait, all participants started at a marked point on the floor 2 meters before the 5-meter OptoGait recording area, and then walked until they reached a marked point 2 meters beyond the recording area.

To study cognitive-motor interactions, participants were instructed to walk at their preferred speed, while performing a cognitive interference task, which required them to recite alternate letters of the alphabet starting with the letter “A” (Verghese et al., 2010, 2016). They were instructed to pay equal attention to walking and talking to minimize task prioritization effects (Verghese et al., 2007). The number of errors and correctly recited alternate letters were recorded by the research assistants. Each participant completed a baseline practice trial of reciting alternate letters of the alphabet while standing prior to the dual-task walking.

#### Cognitive Measures

Executive function and attention are two essential cognitive resources required for normal walking (Montero-Odasso et al., 2012). The Trail Making Test (TMT) is divided into two parts and provides general information on visual search, scanning, speed of processing, mental flexibility, and executive function (Tombaugh, 2004). Part A assesses simple visual attention and sequencing (Reitan and Wolfson, 1985), while Part B assesses executive functions linked to prefrontal cortex activity (Kubo et al., 2008). Part B was strategically included to detect CCT-related transfer of learning (Marusic et al., 2018a).

#### Electrophysiological and Psychophysical Measures

Scalp electroencephalographic (EEG) activity was recorded using g.tec medical engineering equipment (Schiedlberg, Austria, EU), with 32 Ag/AgCl electrodes, arranged according to the International 10–20 System. The sampling rate was 512 Hz with 32-bit resolution.

Due to time- and study resource-constrains, only a sub-group of 23 intervention and 22 control participants were randomly selected and measured before and after the 2-month study period (see Figure 1). Participants were assessed while seated in a neutral position. After collection of baseline measurements with eyes open and closed, participants were instructed to perform a simple reaction time test in response to 70 visual stimuli presented with a random inter-stimulus interval between 2 and 5 secs on a 17.0-inch flat panel LCD monitor (120-Hz refresh rate) situated ~50 centimeters in front of them. Their task was to press the response button as quickly as possible with the bottom of the right foot (which rested on the response pad). The response pad was connected to a trigger box (g.tec TRIGbox). The visual stimuli were presented in the center of a monitor (circular disc with a 5 cm radius was presented against a black background at the center of the display, duration 150 ms, intensity 50 cd/m^2^) placed directly in front of the participant's field of view. Stimulus-locked ERPs (s-ERPs; also known as visual-evoked potentials VEPs) and response-locked ERPs (r-ERP; for the lower limb, also known as motor-related cortical potentials MRCPs) were extracted as described in Yordanova et al. (2004). The s-ERPs correspond to occipital electrodes (averaged across O1, Oz, and O2) and show visual components P1, N1, and P2 after the visual stimuli occurrence while the r-ERPs (MRCP) correspond to central electrode Cz and show the peak latency and amplitude of the most negative displacement of the MRCP, prior to or at the time of response execution. Details about sensorimotor task and EEG/ERP processing can be found in manuscript of Marusic et al. (2022).

### Statistical Analyses

Data were analyzed in SPSS software version 26.0 (IBM, Chicago, IL). An examination for normal distribution was conducted using the Shapiro-Wilk's test and visual inspection (histogram and Q-Q plot). To examine potential group differences in demographic and clinical outcome measures at baseline, an independent-sample *t*-test was performed. The difference between groups in attrition rate was assessed using chi-square statistics. A mixed linear model (MLM) analysis of variance was used to test the effect of the 2-month CCT intervention on the outcome measures in comparison with the control group. Groups were compared using the intention-to-treat principle with MLM adjusted for confounders (baseline MoCA score, age, and gender). While the MLM can handle missing data due to dropout missing at random (Laird and Ware, 1982), the analysis controlled for attrition in both arms by including a dichotomous ‘study completion' variable as a covariate in the model. More specifically, *Group* (experimental *vs*. control), *time* (pre- *vs*. post-intervention), and *group* × *time interaction* terms were treated as fixed effects and *participants* as a random effect. Maximum Likelihood (ML) was used to produce parameter estimates. The alpha level was set to 0.05.

## Results

### Group Characteristics

Of the 63 participants, 32 were randomized to the CCT and 31 to the control group. Table 1 represents the baseline characteristics of the final sample. The majority of participants were women (78% in CCT and 74% in control group); however, the two groups did not demonstrate significant differences across any key demographic variables suggesting adequate randomization. The sub-sample of 45 participants for EEG/ERP analysis also did not show any group differences in baseline characteristics (all *p*s ≥ 0.125).

**Table 1:** Table of baseline characteristics for intervention and control group (intention to treat).

**Variables**	**Intervention**	**Control**	***p*** **value**
N	32	31	
Age (years)	67.7 ± 5.8	67.2 ± 6.0	0.757
Women (*n*)	25	23	
BMI (kg/m^2^)	25.4 ± 5.3	25.8 ± 4.4	0.725
Education (years)	13.4 ± 2.1	12.9 ± 2.1	0.294
MoCA score (0–30)	27.4 ± 1.7	27.0 ± 1.7	0.353
General health status (1–5)^*^	3.6 ± 0.6	3.7 ± 0.7	0.736
Self-selected gait speed ST(m/s)	1.14 ± 0.21	1.18 ± 0.22	0.356
Self-selected gait speed DT (m/s)	0.93 ± 0.25	0.95 ± 0.23	0.873
TMT A (sec)	41.8 ± 19.7	46.6 ± 23.5	0.425
TMT B (sec)	98.9 ± 50.1	99.2 ± 52.4	0.987

* *1, minimal; 5, best; BMI, body mass index; MoCA, Montreal Cognitive Assessment; TMT, Trail Making Test*.

The attrition in the control group (*n* = 9) was significantly higher than the attrition in the intervention group (*n* = 3; (χ_(1)_ = 3.946, *p* = 0.047). In the control group, 3 participants were lost due to medical issues and 6 participants did not show up for the POST measurement session. In the intervention group, 1 participant was lost due to medical issues and 2 participants discontinued the intervention program (see also Figure 1). There were no significant differences in baseline characteristics parameters between those who dropped vs. those who completed the study (*p* ≥ 0.217); however, we included study completeness as a covariate in the MLM. In the intervention group, the CCT adherence was high (87.3%). When queried about self-reported daily computer exposure, eight CCT participants (25.0%) reported daily computer exposure prior to the study, 12 (37.5%) reported moderate exposure, while the remaining 12 (37.5%) reported minimal to no computer exposure.

### Primary Outcomes

Primary outcome results are presented in Table 2.

**Table 2:** Mixed-effect linear model results for primary outcome measures revealing Group, Time, and Group × time interactions as well as adjustments.

**Parameter**	**Estimate**	**Std. error**	**df**	***t***	**Sig**.	**95% confidence interval**	
						**Lower bound**	**Upper bound**
**Self-selected single-task gait speed**							
Group * Time	−0.098	0.050	48.222	−1.949	0.057	−0.199	0.003
Gender	−0.001	0.058	47.871	−0.016	0.987	−0.118	0.116
Age	−0.002	0.005	46.995	−0.332	0.741	−0.011	0.008
MoCA total score	0.008	0.015	47.122	0.529	0.599	−0.023	0.039
Study completion	−0.036	0.072	54.746	−0.500	0.619	−0.179	0.108
**Self-selected dual-task gait speed**							
Group * Time	−0.130	0.046	45.127	−2.790	**0.008**	−0.223	−0.036
Gender	0.016	0.076	47.156	0.212	0.833	−0.136	0.168
Age	−0.007	0.006	46.625	−1.124	0.267	−0.019	0.005
MoCA total score	−0.006	0.020	46.516	−0.315	0.754	−0.046	0.034
Study completion	−0.065	0.091	51.444	−0.714	0.479	−0.248	0.118

*Significant group × time interactions are signed with bold*.

#### Self-Selected Single-Task Gait Speed

At baseline, no difference between group was observed for the self-selected single-task gait speed (*p* = 0.226). However, there was a non-significant group × time interaction trend (*p* = 0.057; Table 2). *Post-hoc* analyses revealed that self-selected gait speed significantly increased within the intervention group (+17.0 ± 21.3%; *p* <0.001), while there was a lesser within group improvement for the control group (+7.2 ± 15.4%; *p* = 0.096). Comparison of speed in the self-selected single-task gait speed condition showed that older adults in the intervention group walked 0.16 m/s faster at the end of the CCT intervention, whereas participants in the control group increased their speed by 0.07 m/s from baseline.

#### Self-Selected Dual-Task Gait Speed

At baseline, no difference between group was observed for the self-selected dual-task gait speed (*p* = 0.901). The MLM revealed a significant group × time interaction (*p* = 0.008) indicating that CCT improved dual-task performance in the intervention compared to the control group. *Post-hoc* analyses revealed that self-selected dual-task gait speed significantly increased within the intervention group (+20.6 ± 24.8%; *p* < 0.001), while there were no within group changes pre- to post-intervention for the control group (+3.0 ± 13.8%; *p* = 0.467). Older adults in the intervention group walked 0.13 m/s faster in self-selected dual-task gait speed condition at the end of the CCT intervention, whereas participants in the control group increased their speed by 0.02 m/s from baseline.

### Secondary Outcomes

Secondary outcome results are presented in Table 3. We had reported blood draws as secondary outcomes in the clinicaltrials.gov registration form, but these are not included in this manuscript due to their complexity.

**Table 3:** Mixed-effect linear model results for secondary outcome measures revealing Group, Time and Group × time interactions as well as covariates.

**Parameter**	**Estimate**	**Std. error**	**df**	***t***	**Sig**.	**95% confidence interval**	
						**Lower bound**	**Upper bound**
**TMT A**							
Group * Time	6.999	4.625	44.007	1.513	0.137	−2.322	16.320
Gender	−6.746	5.808	48.420	−1.161	0.251	−18.422	4.930
Age	−0.109	0.484	50.718	−0.225	0.823	−1.082	0.864
MoCA total score	−0.986	1.510	47.039	−0.653	0.517	−4.024	2.052
Study completion	2.269	7.295	61.307	0.311	0.757	−12.317	16.855
**TMT B**							
Group * Time	28.063	11.981	42.796	2.342	**0.024**	3.898	52.227
Gender	−19.074	12.426	46.818	−1.535	0.132	−44.075	5.926
Age	0.628	1.042	49.984	0.603	0.550	−1.465	2.720
MoCA total score	−8.219	3.220	45.026	−2.552	0.014	−14.705	−1.733
Study completion	8.984	16.053	65.965	0.560	0.578	−23.067	41.034
**Foot reaction times**							
Group * Time	27.685	11.034	25.641	2.509	**0.019**	4.988	50.382
Gender	17.642	14.572	33.320	1.211	0.235	−11.994	47.277
Age	1.399	1.100	31.922	1.272	0.213	−0.842	3.639
MoCA total score	−1.408	3.991	33.024	−0.353	0.727	−9.528	6.713
Study completion	4.755	15.369	38.974	0.309	0.759	−26.332	35.841
**Stimulus processing P1 amplitude**							
Group * Time	1.301	1.069	18.550	1.218	0.239	−0.939	3.542
Gender	1.604	0.817	22.222	1.962	0.062	−0.090	3.298
Age	0.135	0.073	26.747	1.849	0.076	−0.015	0.285
MoCA total score	−0.556	0.223	21.098	−2.493	0.021	−1.020	−0.092
Study completion	−0.311	1.194	33.987	−0.260	0.796	−2.736	2.115
**Stimulus processing P1 latency**							
Group * Time	6.558	9.866	17.900	0.665	0.515	−14.177	27.294
Gender	−11.821	8.788	23.016	−1.345	0.192	−30.000	6.357
Age	0.652	0.772	26.575	0.845	0.406	−0.932	2.236
MoCA total score	2.947	2.408	22.256	1.224	0.234	−2.044	7.938
Study completion	1.557	11.839	31.465	0.132	0.896	−22.574	25.689
**Stimulus processing N1 amplitude**							
Group * Time	−1.112	1.073	16.109	−1.036	0.315	−3.385	1.161
Gender	−1.666	1.329	23.356	−1.253	0.223	−4.414	1.082
Age	−0.089	0.114	25.436	−0.782	0.441	−0.323	0.145
MoCA total score	0.578	0.366	23.007	1.578	0.128	−0.180	1.335
Study completion	−1.160	1.420	22.980	−0.817	0.423	−4.097	1.778
**Stimulus processing N1 latency**							
Group * Time	12.160	12.512	18.091	0.972	0.344	−14.117	38.438
Gender	−11.126	10.145	22.389	−1.097	0.284	−32.144	9.892
Age	0.166	0.900	26.572	0.185	0.855	−1.681	2.014
MoCA total score	2.400	2.774	21.415	0.865	0.397	−3.362	8.161
Study completion	6.386	14.400	33.431	0.443	0.660	−22.896	35.668
**Stimulus processing P2 amplitude**							
Group * Time	−0.899	1.218	14.437	−0.738	0.472	−3.505	1.706
Gender	2.330	1.497	21.813	1.556	0.134	−0.777	5.437
Age	−0.039	0.128	24.097	−0.302	0.765	−0.303	0.226
MoCA total score	−0.328	0.412	21.433	−0.795	0.435	−1.184	0.528
Study completion	1.374	1.610	21.597	0.854	0.403	−1.968	4.716
**Stimulus processing P2 latency**							
Group * Time	27.012	8.656	13.046	3.121	**0.008**	8.320	45.704
Gender	−0.851	13.851	21.462	−0.061	0.952	−29.619	27.917
Age	−1.726	1.173	22.911	−1.471	0.155	−4.153	0.702
MoCA total score	−1.234	3.821	21.246	−0.323	0.750	−9.174	6.707
Study completion	16.787	11.944	16.752	1.405	0.178	−8.442	42.016
**Motor-related processes: peak amplitude**							
Group * Time	−3.501	1.207	18.500	−2.901	**0.009**	−6.032	−0.971
Gender	1.380	1.279	24.186	1.079	0.291	−1.259	4.018
Age	0.006	0.109	23.468	0.059	0.953	−0.219	0.232
MoCA total score	−0.097	0.342	24.113	−0.283	0.780	−0.802	0.609
Study completion	1.924	1.596	30.938	1.206	0.237	−1.330	5.178
**Motor-related processes: peak latency**							
Group * Time	−0.549	13.023	14.251	−0.042	0.967	−28.435	27.337
Gender	−3.477	13.640	20.750	−0.255	0.801	−31.864	24.909
Age	−2.026	1.642	19.614	−1.233	0.232	−5.456	1.405
MoCA total score	−2.971	3.669	20.245	−0.810	0.428	−10.618	4.677
Study completion	−7.629	17.608	15.684	−0.433	0.671	−45.017	29.759
**Motor-related processes: onset latency**							
Group * Time	−19.049	21.215	14.570	−0.898	0.384	−64.385	26.287
Gender	5.535	16.212	17.792	0.341	0.737	−28.555	39.624
Age	−1.108	1.986	16.088	−0.558	0.584	−5.316	3.099
MoCA total score	−5.727	4.368	17.060	−1.311	0.207	−14.941	3.488
Study completion	9.188	25.816	16.683	0.356	0.726	−45.358	63.735

*Significant group × time interactions are signed with bold*.

#### TMT A

At baseline, no difference between groups was observed in TMT A test (*p* = 0.397). The MLM revealed no significant group × time interaction (*p* = 0.137). Delta values showed that post-CCT, older adults in the intervention group solved TMT A, on the average, 9.8 s faster compared to older adults in the control group who solved TMT A, on the average, 2.5 s faster.

#### TMT B

At baseline, no difference between groups was observed in TMT B test (*p* = 0.948). The MLM revealed a significant group × time interaction (*p* = 0.024). *Post-hoc* analyses revealed that time to complete the TMT B was significantly reduced in the intervention group (−21.1 ± 31.1%; *p* = 0.002), while there were no changes pre- to post-intervention for the control group (+2.9 ± 33.0%; *p* = 0.855). Delta values showed that older adults in the intervention group solved TMT B on average 31.9 s faster at the end of the CCT intervention, whereas participants in the control group reduced solving time by 1.3 s.

#### Foot Reaction Time Test

At baseline, no difference between groups was observed in simple reaction time using lower limbs (*p* = 0.932). However, the MLM revealed a significant group × time interaction (*p* = 0.019). *Post-hoc* analyses revealed that simple reaction time was significantly reduced in the intervention group (−5.0 ± 9.9%; *p* = 0.040), however remained unchanged in the control group (+2.7 ± 9.4%; *p* = 0.222) during the pre- to post-intervention period. Delta values showed that older adults in the intervention group had on average foot reaction time 9.5 ms faster at the end of the CCT intervention, whereas participants in the control group prolonged their foot reaction time on average by 6.8 ms.

#### ERPs

**Figure 2:**
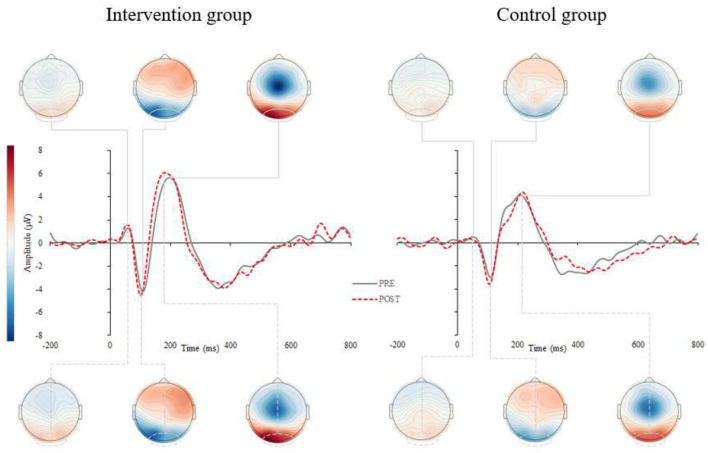
Grand average stimulus-locked event-related potentials (s-ERPs) for the intervention (left) and control (right) group. Figure represents the grand averaged ERPs for pre- (gray, solid) and post-assessments (red, dashed) for intervention (left) and control group (right). The s-ERPs correspond to occipital electrodes (averaged across O1, Oz, and O2) and show visual components P1, N1 and P2 after the visual stimuli occurrence.

**Figure 3:**
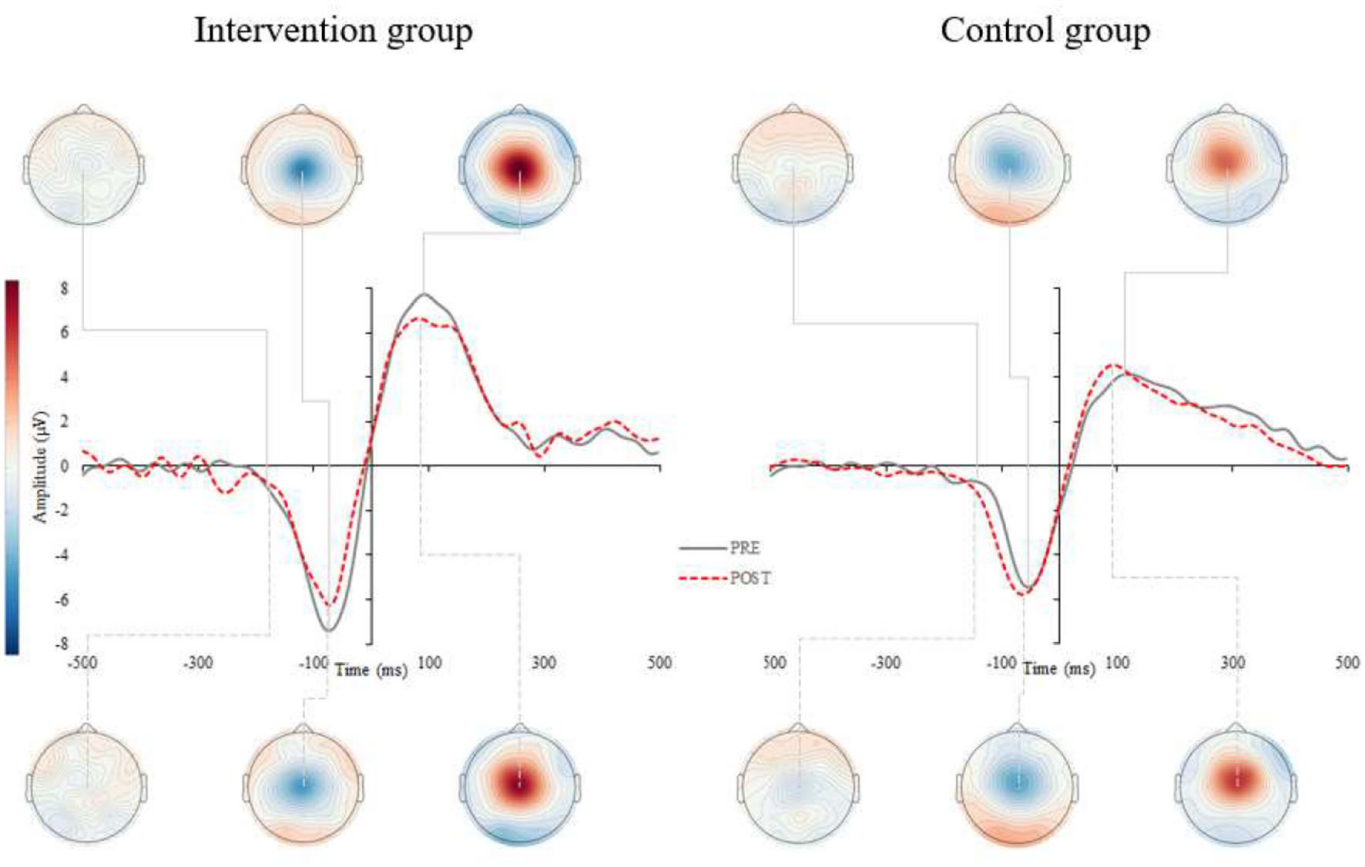
Grand average response-locked event-related potentials (r-ERPs) for the intervention (left) and control (right) group. Figure represents the grand averaged ERPs for pre- (gray, solid) and post-assessments (red, dashed) for intervention (left) and control group (right). The r-ERPs (MRCP) correspond to central electrode Cz and show the peak latency and amplitude of the most negative displacement of the MRCP, prior to or at the time of response execution.

Results for the ERP components are summarized in Figures 2, 3 and Table 2. A significant interaction between *group* and *time* was found for the P2 latency component (*p* = 0.008). *Post-hoc* analyses revealed that P2 latency was significantly reduced in the intervention group (−6.6 ± 5.7%; *p* < 0.001; Figure 2 left), there were no changes pre- to post-intervention for the control group (+1.8 ± 9.1%; *p* = 0.500). Delta values revealed that older adults in the intervention group reduced their P2 latency on average for 14.6 ms at the end of the CCT intervention, whereas participants in the control group prolonged their P2 latency by 4.0 ms.

Similarly, a significant interaction was found for the peak amplitude in motor-related processes (*p* = 0.009). *Post-hoc* analyses revealed that MRCP peak amplitude was significantly reduced in the intervention group (−14.5 ± 23.3%; *p* = 0.029; Figure 3 left), though there were no changes pre- to post-intervention for the control group (+22.6 ± 47.7%; *p* = 0.154). Delta values indicated that older adults in the intervention group reduced their MRCP peak amplitude on average for 1.1 uV at the end of the CCT intervention, whereas participants in the control group increased their MRCP peak amplitude by 0.2 uV. No other significant interactions were found for the remaining ERP components (see Table 3).

#### Correlation Analyses

There were significant correlations found only in the Intervention group between delta scores on TMT B and self-selected dual-task gait speed (r = −0.42; *p* = 0.041), TMT B and P2 latency (r = 0.62; *p* = 0.025), and self-selected dual-task gait speed and P2 latency (r = −0.68; *p* = 0.006).

## Discussion

The findings from this single-blind randomized controlled trial reveal that CCT was effective in improving not only gait but also executive function in active, healthy and independently living older adults. At the conclusion of this structured 2-month cognitive training program (24 sessions of CCT), the intervention group exhibited improved gait performance, especially during challenging walking conditions, relative to the control group. Enhanced primary/gait outcome measures were accompanied with enhanced performance on a test of executive function (TMT) as well as on a sensorimotor task for lower limbs. This was also evident from a correlation analysis showing that improvements in motor and cognitive functions were related to a shortening of the P2 component of visual processing. To our knowledge, this is the first study to reveal that CCT alone can improve complex mobility performance in active, healthy older adults. These results extend previous behavioral reports of the impact of CCT on both cognitive and motor performance in less healthy populations (Mewborn et al., 2017; Marusic et al., 2018b), as well as provide preliminary support for neural enhancements in the aging brain.

The improvement in executive functions in the intervention group supports near transfer of training effects of CCT as demonstrated in previous studies (Klusmann et al., 2010; Heinzel et al., 2014; Marusic et al., 2018a). Compared to the controls, participants in the intervention group required less time to complete the TMT B task - a test of executive functioning that was not used for training purposes in the CogniFit software. Also, the TMT was administered only twice (both pre- and post- study) to both the intervention and control groups, so practice effects across both groups cannot explain enhancements in performance solely for the intervention group. Traditionally, training interventions have only evaluated cognitive training-related improvements in the directly trained cognitive domain (Butler et al., 2018), although some initial cognitive interventions report potential transfer effects to other domains like driving performance and daily functioning (Ball et al., 2002; Willis et al., 2006).

Far transfer, to a distal untrained domain, such as mobility was reported in the present trial in a sample of asymptomatic older adults where self-selected dual-task gait speed was significantly increased (0.13 m/s = 21% intervention *vs*. 0.02 m/s = 3% control improvement) in the intervention compared to control group. Moreover, there was a trend toward improved walking speed during the single-task (0.16m/s = 17% intervention *vs*. 0.07m/s = 7% control improvement). Similar findings were obtained in a pilot study of sedentary older adults (Verghese et al., 2010) and latter summarized in our meta-analytical review (Marusic et al., 2018b) that highlighted larger cognitive training-related improvements during challenging walking conditions given their reliance on higher-order executive functions. From the perspective of clinically meaningful change, both gait speeds were increased by more than 0.08 m/s in the intervention group, which was considered a meaningful change in physical performance (Kwon et al., 2009). Recently, a larger CCT study of 383 non-demented seniors at high risk for mobility disability was completed (Verghese et al., 2021). The authors reported an improvement in walking, but this was no greater than in the active control group, who also completed the same amount of CCT but with no progress in the difficulty of the games performed (Verghese et al., 2021). Here, the authors report that practice effects might explain the improvements seen in both arms and the lack of between-group differences; however, the investigators also discuss an alternate hypothesis that the more robust effect on executive function tests in the cognitive remediation arm compared to the active control may indicate a true training effect, and raises the possibility that even low levels of cognitive remediation (as in their active control arm) lead to cognitive benefits (Verghese et al., 2021). Nevertheless, the current study differed in several ways that might explain the discrepancy in results including, smaller sample size, use of healthy elderly population, wait list control, and the inclusion of neurophysiological measures that were lacking in the previous trial.

The near and far transfer improvements in the current experiment suggest that common brain substrates involving cognitive and mobility processes were strengthened by the CCT. The electrophysiological study in a subgroup of our participants provides further insights into the observed benefits. That is, the intervention group manifested shortened foot reaction times to visual stimuli after 2 months of CCT with concomitant P2 latency reduction over occipital regions as well as decreased MRCP amplitudes over motor cortex region. The former likely reflects enhancements in executive attention processes and sensory encoding (Finnigan et al., 2011), while the latter points toward the optimization in response generation and efficiency in depolarization of motor cortex neurons (Yordanova et al., 2004). While slower foot reaction times have been associated with falls (Lord et al., 2003), a recent study revealed a link between participation in cognitive activities and neuromotor performance (Cai et al., 2020). Although using a cross-sectional design, the latter study showed the participation in cognitively stimulating activities is associated with shorter foot reaction times and faster gait speed (Cai et al., 2020), which is consistent with our longitudinal observations.

Enhancing neural substrates and consequently (sub)components of executive functions [i.e., cognitive flexibility; for details see (Diamond, 2013)] seems like a plausible explanation for improvements in gait (Marusic et al., 2018b), as well as sensory functioning. Previous EEG/ERP studies typically report a u-shape pattern in ERP components (Reuter et al., 2019), and more specifically increased P2 component latencies in older as compared to younger adults (Goodin et al., 1978; Iragui et al., 1993). Our recent study found overall larger amplitudes with delayed latencies of endogenous potentials in older compared to younger adults (Marusic et al., 2022), which is consistent with our recent findings in which P2 latency was significantly reduced after CCT. Moreover, the lower MRCP amplitude during sensorimotor task performed with the lower limbs may indicate less intense depolarization of motor cortex neurons or more efficient recruitment of neuronal resources required for the lower extremity response task after CCT (Yordanova et al., 2004; Marusic et al., 2022).

It is well-documented that walking, a rhythmic motor task, involves complex motor, sensory and cognitive processes (Holtzer et al., 2006; Scherder et al., 2007; Al-Yahya et al., 2011). It is also known that older adults require more attentional demands for motor control while walking, indicating the compounded involvement of attentional resources during gait (Kressig, 2010). Moreover, the association of multisensory integration processes with attention-based performance (Mahoney and Verghese, 2020) and measures of mobility including balance, falls and gait (Mahoney et al., 2019) have been well established. Findings reveal that better performance on a visual-somatosensory simple RT test requiring lower limb responses was associated with better cognitive and motor outcomes in healthy older adults. The authors present a potential overlapping neural circuit which emphasizes the critical role of the prefrontal cortex for intact multisensory, motor and cognitive performance (Mahoney and Verghese, 2020). However, further studies are needed to determine whether CCT simultaneously improves sensory, cognitive, and motor functioning by targeting neural networks with connections to prefrontal cortex.

The main strength of the current study is the application of a randomized control design for examining the effect of CCT. The current study replicates previous studies addressing mobility-related improvements after non-physical training, while revealing the potential of CCT in asymptomatic older adults and highlighting for the first time associated neural alterations.

This study is not without limitations. The sample size was determined by convenience in which we targeted recruitment of older adults attending the Center for daily activities for the elderly. There was a relatively high attrition rate, particularly in the control group, but similar to other cognitive training studies (Ballesteros et al., 2014; Maffei et al., 2017). Attrition rates could likely be improved by including an active control group and/or compensating participants for their time and effort (Verghese et al., 2016). Recent findings by Verghese et al. (2021) in which the active control group showed cognitive and motoric benefits point to the possibility that even low levels of cognitive training have cognitive benefits (Verghese et al., 2021). We were unable to assess durability effects of CCT over time on our outcome measures. Future studies should explore monitoring brain activity directly during actual locomotion through a Mobile Brain/Body Imaging (MoBI) setup for example, to gain further insights into neuroplasticity in ecologically valid environments (Wunderlich et al., 2021).

## Conclusions and Implications

Overall, results from the current study provide evidence that CCT improves mobility in a population of active, healthy and independently living older adults, and is associated with enhancements in cognitive performance. Enhanced executive functions together with optimization of sensorimotor processing that contributes to shorter lower-limb response times after CCT may play a role in enhanced mobility but requires further validation. Our correlational analyses suggest that neurophysiological findings support alterations in neural activity that may contribute to the reported enhancements in cognitive and motor performance. Understanding the underlying mechanisms of how non-physical interventions can improve mobility could guide future research in situations where physical exercise is limited or not possible such as before or after surgery or during periods of immobilization as in hospital stays. There is a need for well-designed large-scale clinical trials with active controls to identify neuronal substrates that are susceptible to CCT enhancement to improve current multimodal interventions to prevent cognitive and motoric declines in aging.

## Data Availability Statement

Raw data supporting the results of this study are available from the corresponding author, upon reasonable request.

## Ethics Statement

All procedures were carried out in accordance with the ethical standards of the 1964 Declaration of Helsinki and were approved by the Republic of Slovenia National Medical Ethics Committee (KME57/06/17).

## Author Contributions

UM: concept creation, project holder, data acquisition monitoring, data processing, data analysis, interpretation of data, and drafted manuscript. JV and JRM: concept creation, interpretation of data, critically reading, and proof-reading the manuscript. All authors read and approved the final manuscript.

## Funding

This study was supported by Slovenian Research Agency (research core Funding No. P5-0381) and European Social Fund and Ministry of Education, Science and Sport (Slovenia, EU). The authors also acknowledge financial support from the European Union's Horizon 2020 research and innovation programme under grant agreement No. 952401 (TwinBrain – TWINning the BRAIN with machine learning for neuro-muscular efficiency).

## Conflict of Interest

JRM has a financial interest in JET Worldwide Enterprises Inc., a digital health startup spun out of research conducted at Albert Einstein College of Medicine and such interest is not related to the current study. The remaining authors declare that the research was conducted in the absence of any commercial or financial relationships that could be construed as a potential conflict of interest.

## Publisher's Note

All claims expressed in this article are solely those of the authors and do not necessarily represent those of their affiliated organizations, or those of the publisher, the editors and the reviewers. Any product that may be evaluated in this article, or claim that may be made by its manufacturer, is not guaranteed or endorsed by the publisher.

## References

[B1] Al-Yahya E. Dawes H. Smith L. Dennis A. Howells K. Cockburn J. (2011). Cognitive motor interference while walking: a systematic review and meta-analysis. Neurosci. Biobehav. Rev. 35, 715–728. 10.1016/j.neubiorev.2010.08.008 20833198

[B2] Azadian E. Majlesi M. Jafarnezhadgero A. A. (2018). The effect of working memory intervention on the gait patterns of the elderly. J. Bodywork Move. Therap. 22, 881–887. 10.1016/j.jbmt.2017.08.008 30368330

[B3] Ball K. Berch D. B. Helmers K. F. Jobe J. B. Leveck M. D. Marsiske M.. (2002). Effects of cognitive training interventions with older adults - A randomized controlled trial. Jama-J. Am. Med. Assoc. 288, 2271–2281. 10.1001/jama.288.18.2271 12425704 PMC2916176

[B4] Ballesteros S. Prieto A. Mayas J. Toril P. Pita C. Ponce de León L.. (2014). Brain training with non-action video games enhances aspects of cognition in older adults: a randomized controlled trial. Front. Aging Neurosci. 6, 277. 10.3389/fnagi.2014.00277 25352805 PMC4196565

[B5] Beauchet O. Allali G. Annweiler C. Verghese J. (2016). Association of motoric cognitive risk syndrome with brain volumes: results from the GAIT study. Journals of Gerontology Series A: Biomedical Sciences and Medical Sciences. 71, 1081–1088. 26946101 10.1093/gerona/glw012

[B6] Blumen H. M. Brown L. L. Habeck C. Allali G. Ayers E. Beauchet O.. (2019). Gray matter volume covariance patterns associated with gait speed in older adults: a multi-cohort MRI study. Brain Imaging and Behavior. 13, 446–460. 29629501 10.1007/s11682-018-9871-7 PMC6177326

[B7] Butler M. McCreedy E. Nelson V. A. Desai P. Ratner E. Fink H. A.. (2018). Does cognitive training prevent cognitive decline?: a systematic review. Ann. Intern. Med. 168, 63–68. 10.7326/M17-1531 29255842

[B8] Cai Y. Hausdorff J. M. Bean J. F. Manor B. You T. Leveille S. G. (2020). Participation in cognitive activities is associated with foot reaction time and gait speed in older adults. Aging Clin. Exp. Res. 3, 1583. 10.1007/s40520-020-01583-3 32415668 PMC9514892

[B9] Cohen R. G. Vasavada A. N. Wiest M. M. Schmitter-Edgecombe M. (2016). Mobility and upright posture are associated with different aspects of cognition in older adults. Front. Aging Neurosci. 8, 257. 10.3389/fnagi.2016.00257 27877123 PMC5099145

[B10] Demnitz N. Zsoldos E. Mahmood A. Mackay C. E. Kivimäki M. Singh-Manoux A.. (2017). Associations between mobility, cognition, and brain structure in healthy older adults. Front. Aging Neurosci. 9, 155. 10.3389/fnagi.2017.00155 28588477 PMC5440513

[B11] Diamond A. (2013). Executive functions. Ann. Rev. Psychol. 64, 135–168. 10.1146/annurev-psych-113011-143750 23020641 PMC4084861

[B12] DiPietro L. Jin Y. Talegawkar S. Matthews C. E. (2018). The joint associations of sedentary time and physical activity with mobility disability in older people: the NIH-AARP diet and health study. J. Gerontol. Series A 73, 532–538. 10.1093/gerona/glx122 28958064 PMC5861886

[B13] Finnigan S. O'Connell R. G. Cummins T. D. Broughton M. Robertson I. H. (2011). ERP measures indicate both attention and working memory encoding decrements in aging. Psychophysiology 48, 601–611. 10.1111/j.1469-8986.2010.01128.x 21039584

[B14] Gajewski P. D. Falkenstein M. (2012). Training-induced improvement of response selection and error detection in aging assessed by task switching: effects of cognitive, physical, and relaxation training. Front. Hum. Neurosci. 6, 130. 10.3389/fnhum.2012.00130 22593740 PMC3349932

[B15] Gajewski P. D. Falkenstein M. (2018). ERP and behavioral effects of physical and cognitive training on working memory in aging: a randomized controlled study. Neural Plasticity 2018, 835. 10.1155/2018/3454835 29796016 PMC5896218

[B16] Goodin D. S. Squires K. C. Henderson B. H. Starr A. (1978). Age-related variations in evoked potentials to auditory stimuli in normal human subjects. Electroencephalogr. Clin. Neurophysiol. 44, 447–458. 10.1016/0013-4694(78)90029-9 76553

[B17] Heinzel S. Schulte S. Onken J. Duong Q.-L. Riemer T. G. Heinz A.. (2014). Working memory training improvements and gains in non-trained cognitive tasks in young and older adults. Aging Neuropsychol. Cogn. 21, 146–173. 10.1080/13825585.2013.790338 23639070

[B18] Hirvensalo M. Rantanen T. Heikkinen E. (2000). Mobility difficulties and physical activity as predictors of mortality and loss of independence in the community-living older population. J. Am. Geriatric. Soc. 48, 493–498. 10.1111/j.1532-5415.2000.tb04994.x 10811541

[B19] Holtzer R. Verghese J. Xue X. Lipton R. B. (2006). Cognitive processes related to gait velocity: results from the Einstein Aging Study. Neuropsychology 20, 215. 10.1037/0894-4105.20.2.215 16594782

[B20] Iragui V. J. Kutas M. Mitchiner M. R. Hillyard S. A. (1993). Effects of aging on event-related brain potentials and reaction times in an auditory oddball task. Psychophysiology 30, 10–22. 10.1111/j.1469-8986.1993.tb03200.x 8416055

[B21] Klusmann V. Evers A. Schwarzer R. Schlattmann P. Reischies F. M. Heuser I.. (2010). Complex mental and physical activity in older women and cognitive performance: a 6-month randomized controlled trial. J. Gerontol. Series a-Biologic. Sci. Med. Sci. 65, 680–688. 10.1093/gerona/glq053 20418350

[B22] Kressig S. A. B. R. W. (2010). Laboratory Review: The Role of Gait Analysis in Seniors' Mobility and Fall Prevention. 20980732 10.1159/000322194

[B23] Kubo M. Shoshi C. Kitawaki T. Takemoto R. Kinugasa K. Yoshida H.. (2008). Increase in prefrontal cortex blood flow during the computer version trail making test. Neuropsychobiology 58, 200–210. 10.1159/000201717 19212135

[B24] Kwon S. Perera S. Pahor M. Katula J. A. King A. C. Groessl E. J.. (2009). What is a meaningful change in physical performance? findings from a clinical trial in older adults (the LIFE-P study). J. Nutr. Health Aging 13, 538–544. 10.1007/s12603-009-0104-z 19536422 PMC3100159

[B25] Laird N. M. Ware J. H. (1982). Random-effects models for longitudinal data. Biometrics 963–974. 10.2307/2529876 7168798

[B26] Lee P. G. Jackson E. A. Richardson C. R. (2017). Exercise prescriptions in older adults. Am. Fam. Physic. 95, 425–432. 28409595

[B27] Lienhard K. Schneider D. Maffiuletti N. A. (2013). Validity of the Optogait photoelectric system for the assessment of spatiotemporal gait parameters. Med. Eng. Physic. 35, 500–504. 10.1016/j.medengphy.2012.06.015 22818403

[B28] Lord S. R. Menz H. B. Tiedemann A. (2003). A physiological profile approach to falls risk assessment and prevention. Phys. Ther. 83, 237–252. 10.1093/ptj/83.3.237 12620088

[B29] Maffei L. Picano E. Andreassi M. Angelucci A. Baldacci F. Baroncelli L.. (2017). Randomized trial on the effects of a combined physical/cognitive training in aged MCI subjects: the Train the Brain study. Sci. Rep. 7, 39471. 10.1038/srep39471 28045051 PMC5206718

[B30] Mahoney J. R. Cotton K. Verghese J. (2019). Multisensory integration predicts balance and falls in older adults. J. Gerontol. Series A 74, 1429–1435. 10.1093/gerona/gly245 30357320 PMC6696711

[B31] Mahoney J. R. Verghese J. (2020). Does cognitive impairment influence visual-somatosensory integration and mobility in older adults? J. Gerontol. Series A 75, 581–588. 10.1093/gerona/glz117 31111868 PMC7328197

[B32] Marusic U. Giordani B. Moffat S. D. Petrič M. Dolenc P. Pišot R.. (2018a). Computerized cognitive training during physical inactivity improves executive functioning in older adults. Aging Neuropsychol. Cogn. 25, 49–69. 10.1080/13825585.2016.1263724 27937138

[B33] Marusic U. Peskar M. De Pauw K. Omejc N. Drevensek G. Rojc B.. (2022). Neural bases of age-related sensorimotor slowing in the upper and lower limbs. Frontiers in Aging Neuroscience. 355. 10.3389/fnagi.2022.819576 PMC9119024 35601618

[B34] Marusic U. Verghese J. Mahoney J. R. (2018b). Cognitive-based interventions to improve mobility: a systematic review and meta-analysis. J. Am. Med. Direct. Assoc. 18, 2. 10.1016/j.jamda.2018.02.002 29680203 PMC6361514

[B35] Mewborn C. M. Lindbergh C. A. Miller L. S. (2017). Cognitive interventions for cognitively healthy, mildly impaired, and mixed samples of older adults: a systematic review and meta-analysis of randomized-controlled trials. Neuropsychol. Rev. 27, 403–439. 10.1007/s11065-017-9350-8 28726168

[B36] Montero-Odasso M. Verghese J. Beauchet O. Hausdorff J. M. (2012). Gait and cognition: a complementary approach to understanding brain function and the risk of falling. J. Am. Geriatr. Soc. 60, 2127–2136. 10.1111/j.1532-5415.2012.04209.x 23110433 PMC3498517

[B37] Nasreddine Z. S. Phillips N. A. Bédirian V. Charbonneau S. Whitehead V. Collin I.. (2005). The Montreal Cognitive Assessment, MoCA: a brief screening tool for mild cognitive impairment. J. Am. Geriatr. Soc. 53, 695–699. 10.1111/j.1532-5415.2005.53221.x 15817019

[B38] Ng T. P. Feng L. Nyunt M. S. Z. Feng L. Niti M. Tan B. Y.. (2015). Nutritional, physical, cognitive, and combination interventions and frailty reversal among older adults: a randomized controlled trial. Am. J. Med. 128, 1225–1236. 10.1016/j.amjmed.2015.06.017 26159634

[B39] Nguyen L. Murphy K. Andrews G. (2019). Cognitive and neural plasticity in old age: a systematic review of evidence from executive functions cognitive training. Ageing Res. Rev. 53, 100912. 10.1016/j.arr.2019.100912 31154013

[B40] Olfers K. J. Band G. P. (2018). Game-based training of flexibility and attention improves task-switch performance: near and far transfer of cognitive training in an EEG study. Psychol. Res. 82, 186–202. 10.1007/s00426-017-0933-z 29260316 PMC5816121

[B41] Pergher V. Wittevrongel B. Tournoy J. Schoenmakers B. Van Hulle M. M. (2018). N-back training and transfer effects revealed by behavioral responses and EEG. Brain Behav. 8, e01136. 10.1002/brb3.1136 30350357 PMC6236237

[B42] Reitan R. M. Wolfson D. (1985). The Halstead-Reitan Neuropsychological Test Battery: Theory and Clinical Interpretation. Tucson, Ariz.: Neuropsychology Press.

[B43] Reuter E.-M. Vieluf S. Koutsandreou F. Hübner L. Budde H. Godde B.. (2019). A Non-linear relationship between selective attention and associated ERP markers across the lifespan. Front. Psychol. 10, 30. 10.3389/fpsyg.2019.00030 30745886 PMC6360996

[B44] Rosano C. Newman A. B. Katz R. Hirsch C. H. Kuller L. H. (2008). Association between lower digit symbol substitution test score and slower gait and greater risk of mortality and of developing incident disability in well-functioning older adults. J. Am. Geriatric. Soc. 56, 1618–1625. 10.1111/j.1532-5415.2008.01856.x 18691275 PMC2631090

[B45] Scherder E. Eggermont L. Swaab D. van Heuvelen M. Kamsma Y. de Greef M.. (2007). Gait in ageing and associated dementias; its relationship with cognition. Neurosci. Biobehav. Rev. 31, 485–497. 10.1016/j.neubiorev.2006.11.007 17306372

[B46] Simons D. J. Boot W. R. Charness N. Gathercole S. E. Chabris C. F. Hambrick D. Z.. (2016). Do “brain-training” programs work? Psychol. Sci. Public Inter. 17, 103–186. 10.1177/1529100616661983 27697851

[B47] Smith-Ray R. L. Hughes S. L. Prohaska T. R. Little D. M. Jurivich D. A. Hedeker D. (2013). Impact of cognitive training on balance and gait in older adults. J. Gerontol. Series B: Psychol. Sci. Soc. Sci. 13, 97. 10.1093/geronb/gbt097 24192586 PMC4542642

[B48] Tardif S. Simard M. (2011). Cognitive stimulation programs in healthy elderly: a review. Int. J. Alzheimers Dis. 2011, 378934. 10.4061/2011/378934 21876829 PMC3157742

[B49] Tombaugh T. N. (2004). Trail making Test A and B: normative data stratified by age and education. Archiv. Clinic. Neuropsychol. 19, 203–214. 10.1016/S0887-6177(03)00039-8 15010086

[B50] Urzi F. Marusic U. Ličen S. Buzan E. (2019). Effects of elastic resistance training on functional performance and myokines in older women—a randomized controlled trial. J. Am. Med. Direct. Assoc. 20, 830–834. 10.1016/j.jamda.2019.01.151 30902674

[B51] Verghese E. Mahoney J. R. Ambrose A. Wang C. Holtzer R. (2016). Cognitive remediation to enhance mobility in older adults: the CREM study. Neurodegenerat. Dis. Manage. 6, 457–466. 10.2217/nmt-2016-0034 27813452 PMC5134757

[B52] Verghese G. Holtzer R. Katz M. Xue X. Buschke H. Pahor M. (2007). Walking while talking: effect of task prioritization in the elderly. Arch Phys Med Rehabil 88, 50–53. 10.1016/j.apmr.2006.10.007 17207675 PMC1894901

[B53] Verghese J. Mahoney J. Ambrose A. F. Wang C. L. Holtzer R. (2010). Effect of cognitive remediation on gait in sedentary seniors. J. Gerontol. Series a-Biologic. Sci. Med. Sci. 65, 1338–1343. 10.1093/gerona/glq127 20643703

[B54] Verghese J. R. Ayers E. Ambrose A. F. Wang C. Holtzer R. (2021). Computerised cognitive remediation to enhance mobility in older adults: a single-blind, single-centre, randomised trial. Lancet Healthy Longev. 2, e571–e579. 10.1016/S2666-7568(21)00173-2 34522910 PMC8437150

[B55] Willis S. L. Tennstedt S. L. Marsiske M. Ball K. Elias J. Koepke K. M.. (2006). Long-term effects of cognitive training on everyday functional outcomes in older adults. JAMA 296, 2805–2814. 10.1001/jama.296.23.2805 17179457 PMC2910591

[B56] Wunderlich A. Vogel O. Šömen M. M. Peskar M. Fricke M. Gramann K.. (2021). Dual-Task Performance in Hearing-Impaired Older Adults-Study Protocol for a Cross-Sectional Mobile Brain/Body Imaging Study. Front. Aging Neurosci. 13, 773287. 10.3389/fnagi.2021.773287 34867299 PMC8633949

[B57] Yordanova J. Kolev V. Hohnsbein J. Falkenstein M. (2004). Sensorimotor slowing with ageing is mediated by a functional dysregulation of motor-generation processes: evidence from high-resolution event-related potentials. Brain 127, 351–362. 10.1093/brain/awh042 14607784

